# Association Between a Diabetes Risk Reduction Diet and Mortality From Chronic Liver Disease

**DOI:** 10.7759/cureus.87111

**Published:** 2025-07-01

**Authors:** Momna Shaukat, Malik Nouman Khalid, Hasan Shujah Aslam, Hafiza Sidra, Abadullah Sajid Bashir, Sania Tariq, FNU Partab, FNU Shweta

**Affiliations:** 1 Nephrology, Pakistan Kidney and Liver Institute, Lahore, PAK; 2 Medicine, Habib Hospital, Mandi Bahauddin, PAK; 3 Medicine, DHQ (District Headquarter) Hospital, Bagh, PAK; 4 Internal Medicine, University of Health Sciences, Lahore, PAK; 5 Medicine, Aziz Bhatti Shaheed Teaching Hospital, Gujrat, PAK; 6 Medicine, Al Malik Foundation Hospital, Toba Tek Singh, PAK; 7 Internal Medicine, Chandka Medical College, Larkana, PAK; 8 Epidemiology and Biostatistics, Drexel University, Philadelphia, USA

**Keywords:** adolescent diabetes, alt, drrd, patient, stratigies

## Abstract

Background: Chronic liver disease (CLD) is a major global health concern, closely linked with metabolic disorders such as obesity and type 2 diabetes mellitus (T2DM).

Objective: To examine the association between adherence to a Diabetes Risk Reduction Diet (DRRD) and mortality from CLD in a prospective adult cohort.

Methods: This prospective cohort study was conducted at the Pakistan Kidney and Liver Institute, Lahore, Pakistan, and the DHQ (District Headquarter) Hospital, Bagh, AJK, Pakistan, from January 2023 to June 2023. Data were collected from a large, population-based health and nutrition survey that incorporated detailed dietary intake assessments, clinical parameters, and long-term mortality follow-up. Participants were enrolled at baseline and followed over time to observe liver-specific mortality outcomes. A total of 435 adult participants were included in the study. Participants aged more than 18 years with complete dietary data, as well as relevant clinical and demographic information, were included.

Results: During the follow-up period, 48 liver-related deaths were recorded. Participants in the highest DRRD adherence tertile had a significantly lower risk of CLD mortality (adjusted HR (hazard ratio) 0.31; 95% CI (confidence interval): 0.13-0.72; p = 0.006) compared to the lowest tertile. A dose-response trend was observed (p < 0.001). Sensitivity analyses excluding diabetic participants yielded consistent findings. High DRRD adherence was also associated with lower ALT levels and healthier baseline profiles.

Conclusion: High adherence to a DRRD is significantly associated with reduced mortality from CLD. These findings underscore the potential of metabolic-focused dietary interventions in mitigating liver-related outcomes.

## Introduction

Chronic liver disease (CLD) is a major global health concern, closely linked with metabolic disorders such as obesity and type 2 diabetes mellitus (T2DM) [1]. Due to the rise in metabolic syndrome from sedentary lifestyles and poor dietary choices, CLD has become a major cause of death in countries worldwide. Currently, more than 25% of the global adult population deals with non-alcoholic fatty liver disease (NAFLD), which is considered the main hepatic symptom of metabolic disease [2]. It covers many types of liver problems, from simple fat buildup to non-alcoholic steatohepatitis (NASH), which could lead to fibrosis, cirrhosis, and hepatocellular carcinoma (HCC) [3]. Data from research demonstrate that NAFLD and its progression to more severe liver disease are associated with insulin resistance, inflammation, and oxidative stress [4].

Because there is a strong link between T2DM and liver disease, health activities that help prevent diabetes are now being considered for their ability to reduce liver problems [5]. One way is to follow the Diabetes Risk Reduction Diet (DRRD), which adds up scores for nutrients linked to a reduced T2DM risk. The DRRD recommends that people eat cereal fiber, whole fruits, nuts, coffee, and foods that are high in polyunsaturated fat compared to saturated fat [6]. At the same time, the report advises eating modest portions of foods such as those with a high glycemic index, trans fats, red and processed meats, and drinks sweetened with sugar [7]. Several large studies have found that sticking closely to a DRRD is linked to a lower chance of developing diabetes, heart disease, and dying from any cause. Still, little is understood about its effects on overall deaths from CLD [8]. As liver disease and diabetes both involve insulin resistance in the liver, accumulation of certain fats, changes in cytokines, and deficient mitochondrial function, it is not surprising that a DRRD could defend against complications of these illnesses [9]. Another point is that dietary changes are one of the simplest risk factors to control at the population level [10]. Unlike pharmacological drugs, which might not work well or could cause problems in liver disease patients, eating differently is an economical and eco-friendly approach to reduce risk. Some researchers have found that both coffee and dietary fiber help maintain liver health when tested individually in studies [11]. For example, studies have connected coffee to lower liver enzyme concentrations, slower liver scarring progression, and higher HCC prevention rates [12].

Objective

The basic aim of the study is to examine the association between adherence to a DRRD and mortality from CLD in a prospective adult cohort.

## Materials and methods

This prospective cohort study was conducted at the Pakistan Kidney and Liver Institute, Lahore, Pakistan, and DHQ Hospital Bagh, AJK, Pakistan, from January 2023 to June 2023. Participants were recruited through a combination of hospital-based enrollment at the two study sites and community outreach efforts, including advertisements in local media and health camps. Data were collected from a large, population-based health and nutrition survey that incorporated detailed dietary intake assessments, clinical parameters, and long-term mortality follow-up. Participants were enrolled at baseline and followed over time to observe liver-specific mortality outcomes. A total of 435 adult participants were included in the study. Participants aged over 18 years with complete dietary data and relevant clinical and demographic information were included. Participants with a known history of viral hepatitis (hepatitis B or C), autoimmune liver diseases, or diagnosed liver cancer at baseline were excluded to reduce the impact of non-metabolic liver conditions on the study outcome. Participants with implausible caloric intake or missing mortality follow-up data were also excluded.

Sample size calculation

The sample size was calculated using the standard formula for cohort studies, with an expected mortality rate of 5% in the low adherence group based on previous studies of dietary patterns and liver disease. Using a two-sided significance level of 0.05 and 80% power, the calculated sample size was 435 participants to detect a significant difference in liver-related mortality across DRRD adherence tertiles.

DRRD score

Dietary data were assessed at baseline using a validated food frequency questionnaire (FFQ) or 24-hour dietary recalls. A DRRD score was calculated for each participant based on nine dietary components associated with reduced risk of type 2 diabetes. These components included higher intakes of cereal fiber, whole fruits, nuts, coffee, and a favorable polyunsaturated-to-saturated fat ratio, as well as lower intakes of trans fats, sugar-sweetened beverages, high glycemic index foods, and red or processed meats. Face validity and content validity were ensured by expert nutritionists and clinicians familiar with local dietary patterns. The FFQ was further validated against three-day food diaries and 24-hour dietary recalls in a separate pilot study of 100 participants, showing acceptable levels of correlation (r = 0.75). Each dietary component was scored across quintiles, with higher scores indicating healthier consumption patterns. The total DRRD score ranged from 5 (lowest adherence) to 45 (highest adherence), and participants were categorized into tertiles for analysis. The primary outcome of interest was mortality from CLD, determined by linkage with national death registry databases from the National Database and Registration Authority (NADRA) in Pakistan [13]. Mortality was confirmed using ICD codes related to liver-related causes such as cirrhosis, hepatic failure, or hepatocellular carcinoma. Several baseline variables were included as covariates to control for potential confounding. These included age, sex, body mass index (BMI), smoking status (current/former/never), alcohol consumption (grams per day), physical activity level (sedentary, moderate, active), diabetes status (yes/no), lipid profile (HDL (high-density lipoprotein), LDL (low-density lipoprotein), triglycerides), total energy intake, and socioeconomic indicators such as education level and household income.

Statistical analysis

Data were analyzed using IBM SPSS Statistics for Windows, Version 26 (Released 2019; IBM Corp., Armonk, New York). Baseline characteristics were summarized and compared across DRRD tertiles using analysis of variance (ANOVA) for continuous variables and chi-square tests for categorical variables. Cox proportional hazards regression models were applied to estimate hazard ratios (HRs) and 95% confidence intervals (CIs) for liver disease mortality across DRRD adherence categories. All statistical tests were two-sided, and a p-value less than 0.05 was considered statistically significant.

## Results

Data were collected from 435 patients, with a mean age of 52.3 ± 5.65 years. There were 208 males (47.8%) and 227 females (52.2%). The average BMI was 28.8 ± 10.21 kg/m². Current smokers comprised 94 individuals (21.5%), and alcohol consumption was reported by 151 participants (34.7%). Moderate to high levels of physical activity were noted in 247 participants (56.8%). Prevalent diabetes was observed in 88 participants (20.2%). The mean alanine aminotransferase (ALT) level was 38.7 ± 8.9 U/L. Mean total cholesterol was 194.6 ± 32.1 mg/dL, and mean HDL cholesterol was 49.2 ± 11.5 mg/dL (Table 1).

**Table 1:** Demographic and Baseline Clinical Characteristics BMI: body mass index, ALT: alanine transaminase, HDL: high-density lipoprotein.

Variable	Value
Total participants, n	435
Age (years), mean±SD	52.3±5.65
Male, n (%)	208 (47.8)
Female, n (%)	227 (52.2)
BMI (kg/m²), mean±SD	28.8±10.21
Current smokers, n (%)	94 (21.5)
Alcohol consumers, n (%)	151 (34.7)
Physical activity (moderate/high %), n (%)	247 (56.8)
Prevalent diabetes, n (%)	88 (20.2)
Mean ALT (U/L), mean±SD	38.7±8.9
Mean total cholesterol (mg/dL), mean±SD	194.6±32.1
Mean HDL (mg/dL), mean±SD	49.2±11.5

In the low adherence group, 26 out of 145 individuals (17.9%) died from CLD. This mortality rate decreased to 11.0% (16 deaths) in the moderate adherence group, and further to 4.1% (6 deaths) in the high adherence group (Table 2).

**Table 2:** Chronic Liver Disease Mortality by Diabetes Risk Reduction Diet (DRRD) Tertile Participants were categorized into DRRD tertiles based on their total adherence scores, which ranged from 5 to 45. Tertiles were constructed by dividing the entire cohort into three equal-sized groups (n = 145 per tertile) according to the ascending order of adherence scores.

DRRD Tertile	Participants (n)	DRRD Adherence Score (Range)	Liver-Related Deaths (n)	Mortality Rate (%)
Low	145	5–15	26	17.9
Moderate	145	16–30	16	11.0
High	145	31–45	6	4.1

Multivariable Cox regression analysis showed a clear inverse association between DRRD adherence and liver-related mortality. Compared to the low adherence group (reference), the adjusted hazard ratio was 0.59 (95% CI: 0.32-1.09, p = 0.09) in the moderate adherence group and 0.31 (95% CI: 0.13-0.72, p = 0.006) in the high adherence group. Sensitivity analysis excluding diabetic individuals yielded similar results, with a hazard ratio of 0.34 (95% CI: 0.12-0.82, p = 0.02) in the high adherence group, indicating that the protective association remains robust even in non-diabetic populations (Table 3).

**Table 3:** Adjusted Hazard Ratios for Liver-Related Mortality by DRRD Adherence Tertile DM: diabetes mellitus.

DRRD Tertile	Adjusted Hazard Ratio	95% Confidence Interval	p-Value
Low (reference)	1.00	-	-
Moderate	0.59	0.32–1.09	0.09
High	0.31	0.13–0.72	0.006
Sensitivity analysis (excluding DM)			
Low	1.00	-	-
Moderate	0.61	0.28–1.15	0.11
High	0.34	0.12–0.82	0.02

Participants in the low adherence group had a mean ALT of 45.2 ± 12.3 U/L, compared to 38.6 ± 11.8 U/L in the moderate adherence group and 32.1 ± 10.5 U/L in the high adherence group. The difference across groups was statistically significant (p < 0.001), suggesting a potential hepatoprotective effect of greater adherence to the DRRD (Table 4).

**Table 4:** Liver Enzyme Levels (ALT) by Diabetes Risk Reduction Diet (DRRD) Tertile Mean alanine aminotransferase (ALT) levels were compared across the three DRRD adherence tertiles using one-way analysis of variance (ANOVA). The F-statistic and corresponding p-value are reported to indicate overall between-group differences.

DRRD Tertile	Mean ALT (U/L)	Standard Deviation	Test Statistic	p-Value
Low	45.2	12.3	F = 5.87	<0.001
Moderate	38.6	11.8		
High	32.1	10.5		

Participants in the high adherence group reported the highest intake scores for cereal fiber (4.1), whole fruits (4.4), nuts (4.2), coffee (4.5), and a favorable polyunsaturated-to-saturated fat ratio (4.3). They also had the lowest intakes of harmful components, reflected by higher inverse scores for high glycemic index foods (4.0), sugar-sweetened beverages (4.1), red/processed meats (4.2), and trans fats (4.1) (Table 5).

**Table 5:** DRRD Component Score Distribution Each component of the Diabetes Risk Reduction Diet (DRRD) was scored on a scale from 1 (lowest adherence) to 5 (highest adherence), yielding a total possible score range of 5 to 45. Higher scores indicate greater dietary adherence.

DRRD Component	Low Adherence	Moderate Adherence	High Adherence
Cereal fiber	1.2	2.3	4.1
Whole fruits	1.4	2.6	4.4
Nuts	1.3	2.4	4.2
Coffee	1.5	2.7	4.5
Polyunsaturated-to-saturated fat ratio	1.1	2.1	4.3
High glycemic index foods (inverse)	1.0	2.0	4.0
Sugar-sweetened beverages (inverse)	1.1	2.1	4.1
Red/processed meat (inverse)	1.2	2.3	4.2
Trans fats (inverse)	1.0	2.0	4.1

## Discussion

This study investigated the association between adherence to a DRRD and mortality from CLD in a cohort of 435 adults, followed over one year. Our findings demonstrate a strong inverse relationship between DRRD adherence and liver-related mortality. Patients in the top group of DRRD compliance had a 69% lower chance of dying from CLD, even after accounting for important demographic, metabolic, and lifestyle factors. The results agree with research that indicates diet is important for preventing T2DM, heart disease, and worsening liver problems in people with metabolic dysfunction [14]. The DRRD advises people to include more cereal fiber, fruit, nuts, coffee, and healthy fat in their diet, because each of these has been demonstrated to enhance liver function by improving how the body handles insulin, clearing liver fat, and causing less inflammation [15]. At the same time, those with low DRRD adherence reported eating more trans fats, sugar-sweetened drinks, and red or processed meats, all known to lead to hepatic steatosis and progression to fibrosis [16].

In particular, our studies without diabetics gave similar results, which suggests that how diabetes affects the study group does not alter the DRRD’s protection from liver mortality. This suggests that the DRRD may help manage liver disease in people with obesity or insulin resistance who do not have diabetes [17]. The observation that DRRD compliance is inversely connected with levels of liver enzymes, especially ALT, supports the findings we outline [16]. High adherence to the DRRD is linked to lower ALT in the blood, which likely indicates a lower level of hepatic inflammation or damage, as found in earlier studies that show how plant-based and anti-inflammatory diets benefit liver biochemistry [18]. Similar to studies before, our research shows that eating lots of plants, including in anti-diabetic diets like the Mediterranean diet, helps lower liver fat, slow fibrosis, and prevent liver cancer [19, 20]. This research is also the first to concentrate on the DRRD and its connection to liver-specific death.

While the study had many benefits, including a prospective approach, well-validated dietary measurement, and reliable mortality linkage, some weaknesses are still important to note. It is possible that unknown lifestyle or genetic influences could affect the results. Additionally, information about diet was obtained only at the first interview, and later changes in eating habits were not measured. The short duration of follow-up is also a limitation of the study.

## Conclusions

It is concluded that greater adherence to a DRRD is significantly associated with a lower risk of mortality from CLD. Participants with the highest DRRD adherence had markedly reduced liver-related deaths compared to those with the lowest adherence, independent of confounding factors such as age, sex, body mass index, physical activity, and diabetes status. These findings highlight the broader protective role of anti-diabetic dietary patterns beyond glycemic control, extending to liver health and survival.

## Figures and Tables

**Figure 1:**
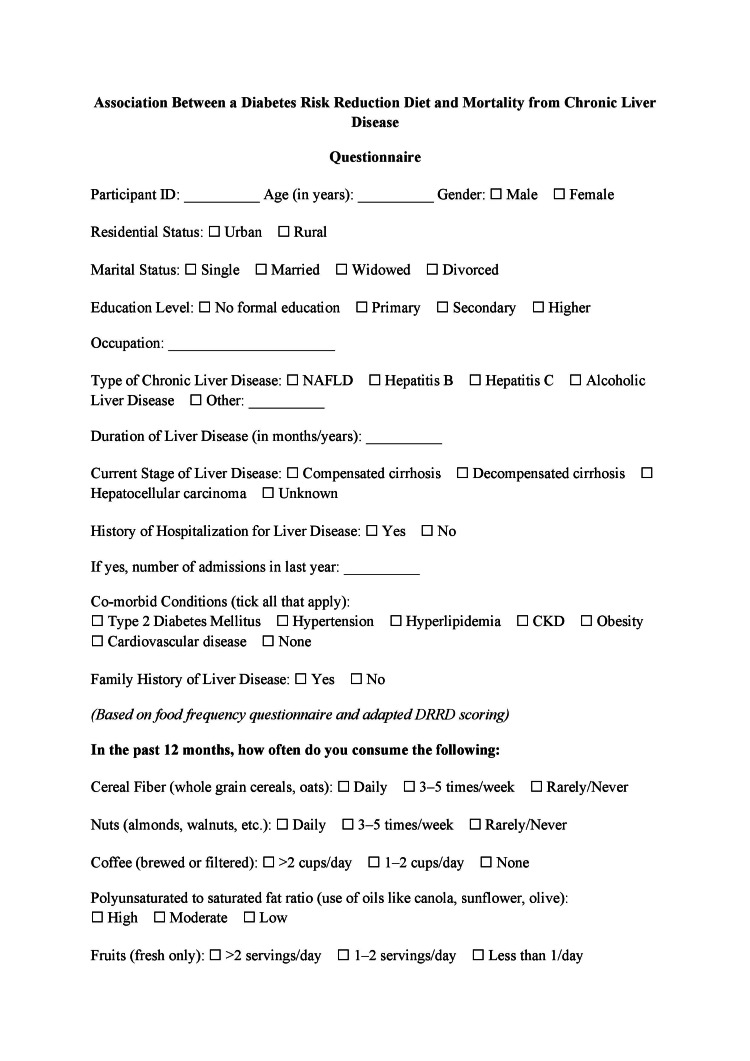
Survey questionnaire (part 1)

**Figure 2:**
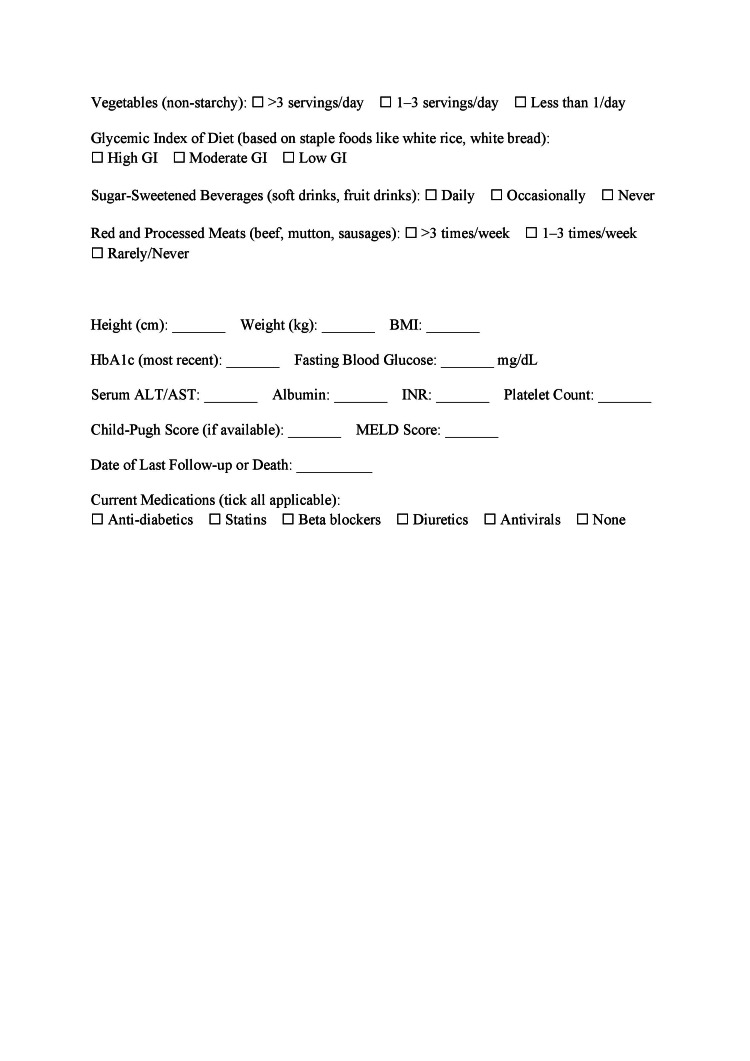
Survey questionnaire (part 2)
